# The Blinded-Dose Purchase Task: assessing hypothetical demand based on cocaine, methamphetamine, and alcohol administration

**DOI:** 10.1007/s00213-023-06334-6

**Published:** 2023-03-04

**Authors:** Meredith S. Berry, Gideon P. Naudé, Patrick S. Johnson, Matthew W. Johnson

**Affiliations:** 1 Behavioral Pharmacology Research Unit, Department of Psychiatry and Behavioral Sciences, Johns Hopkins University School of Medicine, 5510 Nathan Shock Drive, Baltimore, MD 21224 USA; 2 Department of Health Education and Behavior, University of Florida, Yon Hall Room 031, Gainesville, FL 32611 USA

**Keywords:** Cocaine, Methamphetamine, Alcohol, Behavioral economics, Demand curve, Purchase task, Blinded-Dose Purchase Task, Drug use, Abuse liability, Unit price, Human

## Abstract

**Rationale:**

Behavioral economic drug purchase tasks quantify the reinforcing value of a drug (i.e., demand). Although widely used to assess demand, drug expectancies are rarely accounted for and may introduce variability across participants given diverse drug experiences.

**Objectives:**

Three experiments validated and extended previous hypothetical purchase tasks by using blinded drug dose as a reinforcing stimulus, and determined hypothetical demand for experienced effects while controlling for drug expectancies.

**Methods:**

Across three double-blind, placebo-controlled, within-subject experiments, cocaine (0, 125, 250 mg/70 kg; *n*=12), methamphetamine (0, 20, 40 mg; *n*=19), and alcohol (0, 1 g/kg alcohol; *n*=25) were administered and demand was assessed using the Blinded-Dose Purchase Task. Participants answered questions regarding simulated purchasing of the blinded drug dose across increasing prices. Demand metrics, subjective effects, and self-reported real-world monetary spending on drugs were evaluated.

**Results:**

Data were well modeled by the demand curve function, with significantly higher intensity (purchasing at low prices) for active drug doses compared to placebo for all experiments. Unit-price analyses revealed more persistent consumption across prices (lower *α*) in the higher compared to lower active dose condition for methamphetamine (a similar non-significant finding emerged for cocaine). Significant associations between demand metrics, peak subjective effects, and real-world spending on drugs also emerged across all experiments.

**Conclusions:**

Orderly demand curve data revealed differences across drug and placebo conditions, and relations to real-world measures of drug spending, and subjective effects. Unit-price analyses enabled parsimonious comparisons across doses. Results lend credence to the validity of the Blinded-Dose Purchase Task, which allows for control of drug expectancies.

**Supplementary Information:**

The online version contains supplementary material available at 10.1007/s00213-023-06334-6.

## Introduction

Behavioral economics offers a broad framework for understanding how the behavior of an organism is maintained by reinforcers (Hursh, 1980, 1984). Behavioral economics is widely applicable to understanding patterns of drug consumption (e.g., effects of price or effort on substance use, relations among various drugs or formulations), and is considered an important framework for abuse liability assessment (Hursh et al., 2005). Behavioral economic drug purchase tasks quantify drug demand (i.e., the relationship between drug price and consumption) and have been used extensively as a measure of drug reinforcer value (Bruner & Johnson, 2014; Johnson & Bickel, 2003; Johnson & Bickel 2006; Johnson et al., 2004; MacKillop et al., 2019; Strickland et al., 2019). By examining multiple metrics (e.g., intensity, rate of change of elasticity), demand analyses offer an advantageous multidimensional evaluation of drug reinforcement compared to traditional approaches that often treat reinforcement as a homogenous concept (Madden & Bickel, 1999; Hursh & Silberberg, 2008; Johnson & Bickel, 2006).

In a standard laboratory preparation, demand curves are generated with fixed-ratio (FR) schedules to model the price of a commodity, often a drug (e.g., Johnson & Bickel, 2006). This method can be expensive and time-consuming. Hypothetical drug purchase tasks, however, allow for a low cost and rapid alternative to evaluate demand curves, and have facilitated the study of demand across laboratory and online delivery with varied populations (see Acuff et al., 2020; Kaplan et al., 2018; MacKillop et al., 2016; Reed et al., 2020; Strickland et al., 2020, for reviews). In these tasks, participants are asked to report the hypothetical amount of drug they would purchase and consume across a range of prices. These tasks have provided systematic data and have been successfully simulated across numerous drugs including heroin (Jacobs & Bickel, 1999), cocaine (Bruner & Johnson, 2014), cannabis (Aston et al., 2015), alcohol (Amlung et al., 2015a, b; Yurasek et al., 2013), and nicotine (Jacobs & Bickel, 1999; MacKillop et al., 2008; Madden & Kalman, 2010; Murphy et al., 2011). To date, the vast majority of purchase tasks have been conducted to assess nicotine and alcohol purchasing.

Simulated purchase tasks have shown relevance for treatment outcomes, dependence levels, substance-related correlates, and individual differences in smoking risk (MacKillop & Murphy, 2007; MacKillop et al., 2016; Nighbor et al., 2020; Zvorsky et al., 2019), as well as sensitivity to pharmacological treatments (McClure et al., 2013), predictive validity (MacKillop, 2016), and test-retest reliability (Strickland et al., 2019). Previous studies have also shown high correspondence between hypothetical and real purchase tasks (Amlung et al., 2012; Amlung & Mackillop, 2015), although some evidence suggests individuals may be more sensitive to increases in price in a real scenario (Amlung & MacKillop, 2015). Wilson et al. (2016) showed cigarettes purchased across prices in hypothetical and potentially real/real outcomes were significantly correlated; however, demand differed somewhat across potentially real/real conditions versus hypothetical outcomes.

Although hypothetical drug purchase tasks allow for more cost- and time-effective methods, and appear to correspond well with real scenarios, most drug purchase task scenarios involve choices about hypothetical drug doses. These tasks require participants to “imagine a standard/average/typical drug dose” which introduces variability across participants given diverse drug expectancy effects and experiences (the expectation of drug effects, variability in potency with typical drugs purchased). Even when the drug dose is defined (Bruner & Johnson, 2014; e.g., a nickel bag, a gram), expectancy issues of drug quality, potency, and purity remain. Outside of the Cigarette Purchase Task (see Bergeria et al., 2019; Cassidy et al., 2019; and Higgins et al., 2018, for examples of experiential simulated purchasing), hypothetical purchase tasks have rarely accounted for such expectancies among individuals who have previous experience with the substance evaluated in the purchase tasks, nor have dose effects been evaluated as the standard. The present series of experiments sought to validate blinded hypothetical purchase tasks in this regard and extend previous research in this area (cf. MacKillop et al., 2019). MacKillop et al. (2019) conducted a study in which stimulant-naïve individuals completed a blinded purchase task following a single dose of D-amphetamine or placebo (MacKillop et al., 2019). Compared to placebo, the single dose of D-amphetamine significantly increased intensity, breakpoint, and *O*_max_, and decreased elasticity, while *O*_max_ and breakpoint mediated the relationship between subjective drug effects and willingness to take the drug again (MacKillop et al., 2019). Therefore, the purpose of the present experiments was to extend this previous work by using drug as a reinforcing stimulus under double-blind conditions among substance users, and assess hypothetical demand for the experienced effects using a hybrid method that controlled for drug expectancy effects. Across three separate experiments, cocaine, methamphetamine, and alcohol were administered and demand was assessed using the Blinded-Dose Purchase Task. Subsequent relations to clinically meaningful variables including abuse liability measures (i.e., subjective effects), as well as self-reported monetary spending on drugs in real-world settings, were evaluated.

## Methods

### Compliance with ethical standards

Johns Hopkins University Institutional Review Board approved these studies, and these studies were performed in accordance with the 1964 Declaration of Helsinki. Participants provided their written informed consent.

### Participants

Secondary data were drawn from three separate experimental laboratory decision-making protocols involving double-blind drug administration of either cocaine hydrochloride (*n* = 12; Johnson et al., 2017), d-methamphetamine hydrochloride (*n* = 19; Berry et al., 2022), or ethyl alcohol (*n* = 25; Johnson et al., 2016). The Johns Hopkins University Institutional Review Board approved all three original studies. All participants were healthy volunteers recruited from Baltimore, MD, and the surrounding areas using newspaper, radio and Internet advertisements, and word of mouth. Eligibility for each of these protocols was determined through an initial phone screening and, upon qualification, individuals were invited for an in-person screening that included general medical, psychiatric, substance use, and sexual history assessments. Table 1 contains demographic characteristics for each group.

**Table 1:** Demographic characteristics across cocaine, methamphetamine, and alcohol studies

Variable	Study		
	Cocaine (*N* = 11)	Methamphetamine (*N* = 17)	Alcohol (*N* = 21)
Age	25.82 (4.05)	27.35 (7.35)	24.43 (2.62)
Sex (*n*, % female)	4 (36.36)	6 (35.29)	9 (42.86)
Race (*n*, %)			
White	7 (63.64)	12 (7.59)	13 (61.90)
Black	2 (18.18)	3 (17.65)	2 (9.52)
Asian	0	0	1 (4.76)
More than one race	2 (18.18)	2 (11.76)	5 (23.81)
Tobacco use (*n*, % smokers)	9 (81.82)	7 (41.18)	5 (23.81)
Education in years	14.86 (1.80)	14.74 (1.93)	15.52 (1.44)
Monthly income	$1695.45 (1662.30)	$1546.18 (913.95)	$1209.52 (1052.69)
Money spent on substance per week in the last month	$58.47 (7.28)	$163.12 (253.86)	$22.76 (23.86)
Estimated number of times substance was used per week in the last month	1.09 (.86)	1.60 (1.24)	3.22 (1.80)
Number of days substance was used in the last month	4.82 (4.31)	6.13 (5.94)	14.64 (8.18)

Variables reported as mean (± *SD*) unless otherwise noted. Sample size values are following outlier and nonsystematic demand screening

#### Cocaine administration study participants

Participation criteria have been reported in detail previously (Johnson et al., 2017), and thus are outlined only briefly here. Inclusion criteria consisted of being 18–45 years of age; could read and understand the consent form; meeting *DSM-IV* criteria for cocaine abuse or dependence; using cocaine during the past month; and to have been using cocaine for at least 1 year. Exclusion criteria involved any history of severe psychiatric disorders; use of any psychiatric medications by prescription; current physical dependence on any drug except for cocaine, caffeine, or nicotine; current interest in entering treatment for substance use; and pregnancy, currently breastfeeding, or not using an effective method of contraception (females only). Individuals were also ineligible to participate if they were susceptible to or had a history of cardiovascular illness or other serious medical disorders.

#### Methamphetamine administration study participants

Participation criteria have been reported in detail previously (Berry et al., 2022). Briefly, to qualify, participants were required to be between 21 and 55 years old; report nonmedical use of stimulants (e.g., cocaine, amphetamine, methamphetamine, methylphenidate) in the past 6 months; be within 20% of their ideal bodyweight according to the Metropolitan Life height/weight table; had sexual intercourse in their lifetime; and be able to read and understand the consent form. Participants were excluded if they were physically dependent on any substance except nicotine or caffeine; were seeking treatment for substance use; were daily prescription users of licit stimulants; had a medical history, physical examination, or laboratory test performed during the screening process revealing any significant illness or other contraindications to methamphetamine administration; were pregnant, nursing, or not using birth control if female; had experienced psychiatric hospitalization in the past 6 months; or had a history of serious head trauma, dementia, or significant cognitive impairment.

#### Alcohol administration study participants

Participation criteria have been reported in detail previously (Johnson et al., 2016). Briefly, participants were recruited using flyers, internet, and newspaper advertisements. Eligible participants were 21–65 years of age; were able to read and understand the consent form; had sexual intercourse in their lifetime; and reported drinking 4–5 drinks per episode at least occasionally. Participants were excluded if they were physically dependent on any substance (excluding nicotine and caffeine); were seeking treatment for substance use; reported a current major psychiatric disorder or psychiatric hospitalization in the past 6 months; or had medical contraindications to alcohol administration. Female participants were excluded if pregnant, nursing, or not using effective contraception.

### General laboratory procedures

Each of these studies consisted of double-blind, randomized, within-subject, placebo-controlled sessions involving nontreatment-seeking individuals. Study duration consisted of three experimental laboratory sessions for the cocaine and methamphetamine groups (placebo and two active doses) and two experimental laboratory sessions for the alcohol group (placebo and one active dose) with no less than 24 h between sessions. Participants were phoned at least 24 h before each scheduled laboratory session and reminded to abstain from using any drugs including alcohol for the day before sessions but they should otherwise maintain their normal routine (e.g., sleep, caffeine consumption). Participants were also asked to refrain from eating or drinking overnight. Scheduled sessions began at 7:00 a.m. and were rescheduled if participants showed nonzero breath alcohol concentration (BrAC) levels. Participants who were regular tobacco smokers were allowed to smoke a cigarette at the start of each day’s session. Participants were then served a standardized low-fat breakfast (2 toast slices or 1 bagel; a single serving of jelly or butter; 355 ml of either apple or grape juice) which was finished by 8:00 a.m.

### Drug administration procedures

For each study, the drugs were prepared in an onsite pharmacy. Subjective drug effects were assessed throughout the session (see primary sources for each study’s time course and additional session timing details). The Blinded-Dose Purchase Task (described in more detail below) was administered at the end of the session so participants could experience the full time course of the drug prior to evaluating how many doses of the experienced drug they would purchase (approximately 2 h and 15 min following cocaine/placebo administration; approximately 7 h and 15 min following methamphetamine/placebo administration; and approximately 5 h and 15 min following completion of alcohol/placebo administration).

#### Cocaine

During three drug administration sessions, participants orally ingested, with water, a size 00 opaque capsule containing either 0 (placebo), 125 mg/70 kg, or 250 mg/70 kg of cocaine hydrochloride. Across sessions, the capsules appeared identical and were each filled completely with a combination of cellulose and/or cocaine hydrochloride. Drug administration was double-blind. Administration order was randomized across participants.

#### Methamphetamine

During three drug administration sessions, participants orally ingested, with water, a size 00 opaque capsule containing either 0 (placebo), 20 mg, or 40 mg of d-methamphetamine hydrochloride (Mylan, Inc.) and cellulose. Across sessions, the capsules appeared identical. Each participant received each condition once across her or his three sessions (i.e., dose conditions were compared within subject). Administration was double-blind, and dose order was counterbalanced.

#### Alcohol

Placebo- and alcohol-containing solutions were prepared in an onsite pharmacy. A weight-based administration procedure was used to determine the volume of a 1 g/kg alcohol dose (USP 95% ethanol; Letco Medical, Decatur, AL) or water to be mixed in grapefruit juice. Total solution volume was determined per bodyweight so that alcohol (or added water) was 8% of solution by volume. This was divided equally across three cups. Participants experienced placebo and alcohol sessions in a pseudo-random order. Each cup was fitted with a lid and a straw with a 95% alcohol-soaked elastic hairband wrapped around it to obscure olfactory discrimination of alcohol and placebo sessions. Drinks were served promptly after being refrigerated at ~4 °C. Participants were instructed to drink 1 cup at a regular pace over the course of a 20-min interval, resulting in a 1-h administration period for all three cups.

### Assessments

#### Substance use patterns and subjective drug effects

Information about individual patterns of substance use was obtained during the in-person screening and the following three variables were selected for analysis for insight into real-world use and spending behavior: (1) the average amount of money participants spent on the substance (or drug class) per week in the last month; (2) the number of days participants used the substance in the last month; and (3) the estimated number of times participants used the substance per week in the last month. To assess within-session subjective drug effects for each blinded dose, at regular post-administration intervals, participants were asked to rate on a 5-point scale, among other characteristics of the dose consumed (see Berry et al., 2022; Johnson et al., 2016, 2017), whether they “liked” the dose they experienced that morning and whether the dose had produced any “good effects” (0 = *not at all*; 1 = *possibly mild, but not sure*; 2 = *definitely mild*; 3 = *moderately*; 4 = *strongly*). For the purposes of the present investigation, we focused on peak subjective effects, defined as the maximum value observed over the time course for each participant.

#### The Blinded-Dose Purchase Task

At the end of each session, participants in the cocaine and methamphetamine groups completed a blinded purchasing task for hypothetical capsules of that morning’s randomized dose (oral placebo, 125 mg/70 kg, or 250 mg/70 kg for cocaine hydrochloride; oral placebo, 20 mg, or 40 mg for d-methamphetamine hydrochloride). The alcohol group completed the Blinded-Dose Purchase Task for drinks associated with either the placebo or 1.0 g/kg dose of ethyl alcohol. In these tasks, participants indicated how many units of that day’s dose they would purchase to consume over the next month across the following 10 escalating price points: $0.01, $0.03, $0.30, $1, $3, $10, $30, $100, $300, and $1000. Price points were presented on separate pages, and were selected primarily to assess the range of purchasing across numerous prices as in previous studies. Participants read the following scenario prior to beginning the purchase task:

Imagine that you have finished the study and will spend the next month in your usual home environment. Also imagine that you have the chance to buy today’s drug dose for your own personal use within the next month. Please consider what you have received today to be a single dose. You can buy as many doses as you like, but you cannot sell, trade, or give them away, and you cannot save them for more than a month. Other than the fact that the doses are for your own use within the next month, there is no limit to the number of doses you can buy. Please do not buy more than you will use...

Participants then responded to the following question for each of the price points: If today’s drug dose costs [X] each, how many drug doses would you buy to use in the next month?

### Data analysis

Orderliness of dose purchasing task data was evaluated according to previously published criteria (Bruner & Johnson, 2014; cf. Stein et al., 2015). Cases were identified as nonsystematic if (1) consumption at one price was greater than consumption at the previous price by more than 20% and (2) consumption greater than 0 was reported after endorsing 0 consumption at a lower price. Responses on the Blinded-Dose Purchase Tasks were analyzed using the exponentiated model of demand (Koffarnus et al., 2015):

1 $$Q=Q_0\times10^{k\left(e^{-\alpha\;\times\left(Q_0\;\times\;C\right)}-1\right)}$$

where *Q* is the quantity of the dose purchased at each price (i.e., *C*), and *Q*_0_ is the maximum quantity purchased at minimal cost. The number of doses purchased for a month at $0.01 was used as *Q*_0_ (hereafter referred to as demand intensity) instead of the model-derived index. The constant *e* represents Euler’s number. The parameter *k* represents the range of consumption in logarithmic units and was calculated by taking the quotient of the log-transformed mean maximum and mean minimum (nonzero) reported consumption values and adding 0.50 (*k* = 4.05 for cocaine; *k* = 1.86 for methamphetamine; and *k* = 2.71 for alcohol); and *α* is the rate of change in elasticity across the entire demand curve. Though nonlinear curves cannot be fit to datasets with 0 or invariant consumption, such data nevertheless provide valuable information about the reinforcing value of a substance. Toward this end, such cases were omitted only from analyses of the *α* parameter. In addition to the Eq. 1 parameters, we calculated three secondary measures for descriptive analyses to evaluate the tenability of this novel purchase task: (1) *P*_max_ is the point of unit elasticity where consumption decreases disproportionately with increases in price (i.e., slope = −1); however, in the present investigation, we determined the price associated with maximum consumption, termed empirical *P*_max_; (2) *O*_max_ is the maximum amount spent on the commodity and is calculated by multiplying *Q* corresponding with *P*_max_ by *P*_max_; (3) finally, breakpoint is the first price to completely suppress consumption. Here, we report breakpoint 1 (*BP*_1_), the last price at which participants indicated they would purchase any doses to account for individuals who reported some level of consumption across all prices.

To conform to distributional assumptions of parametric statistical tests, we performed natural-log (ln) transformations on *α*, *P*_max_, *O*_max_, and *BP*_1_ and square root transformations on intensity and self-reported substance use patterns (e.g., the average amount of money spent on the substance per week in the last month). Separate analyses were conducted for each study. Differences in demand intensity at each of the doses were compared using one-way repeated-measures ANOVAs for the cocaine and methamphetamine studies and a paired *t-*test for the alcohol study. Differences in the *α* parameter between dose conditions were also compared using paired *t*-tests, with analyses restricted to the two active doses experienced in the cocaine and methamphetamine studies. In order to examine the degree to which responses on the purchase task varied as a function of unit price, consumption values for the active doses in the cocaine and methamphetamine studies were subsequently converted to milligrams per 70 kg purchased (cocaine) or milligrams purchased (methamphetamine) by multiplying the units purchased at each price by the dose experienced on that day. Subjective drug effect data were analyzed using one-way repeated-measures ANOVAs for the cocaine and methamphetamine studies and a paired *t*-test for the alcohol study. Bivariate associations between all measures of demand and self-reported substance use patterns were described using Pearson’s *r* and using Spearman’s *ρ* when involving subjective drug effects. Finally, effect sizes for ANOVAs were reported as partial eta squared (${\eta}_{\textrm{p}}^2$) and for *t*-tests as Hedge’s *g*.

## Results

### Modeling drug purchasing behavior

**Fig. 1.**
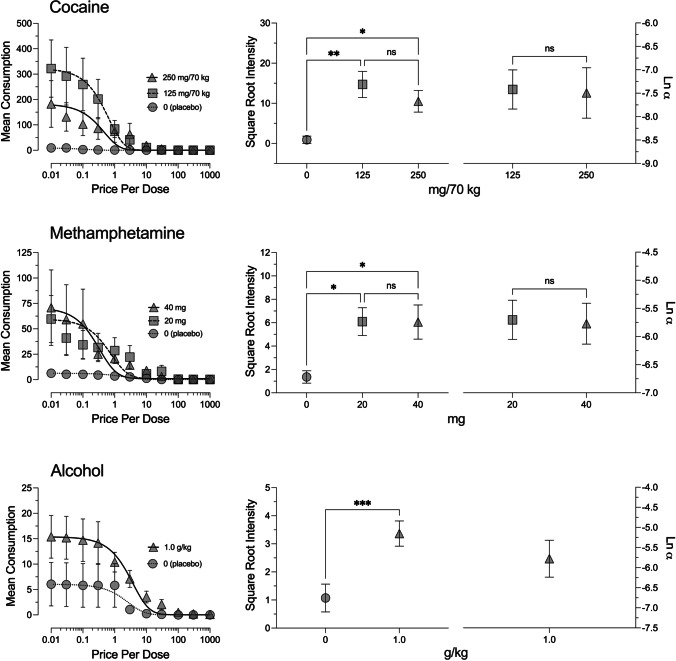
Left panel: Blinded-Dose Purchase Task demand curves for active and placebo doses across the cocaine (top), methamphetamine (middle), and alcohol (bottom) studies. Error bars represent standard error of the mean. Consumption data plotted as a function of price per dose (rather than unit price; see text for additional comparisons based on unit price). Right panel: demand intensity (left *y*-axis) and *α* values (right *y*-axis) across the cocaine, methamphetamine, and alcohol studies. These indices are depicted following square root and natural-log transformations, respectively. All error bars represent standard error of the mean, *p ≤ .05; **p ≤ .01; ***p ≤ .001. Note. For all studies, the high proportion of cases with zero consumption in placebo conditions did not allow for the determination of elasticity parameters. Therefore statistical comparisons of elasticity were restricted to comparison between active doses, which was only possible in the cocaine and methamphetamine studies

Simulated purchasing of each day’s dose varied as an inverse function of increases in price across the three studies (see Fig. 1). Consumption data from the cocaine study were described well by Eq. 1 across the placebo (*R*^2^ = .94, *RMSE* = 0.86), 125 mg/70 kg dose (*R*^2^ = .99, *RMSE* = 13.31), and, to a moderately lesser extent, the 250 mg/70 kg dose (*R*^2^ = .73, *RMSE* = 31.67). Data from one participant were identified as nonsystematic based on the first criterion and excluded from further analyses. There was a significant main effect of dose on demand intensity (*F*_2,20_ = 11.23, *p* < .001, ${\eta}_{\textrm{p}}^2$ = .53), with significantly fewer doses purchased in the placebo condition than in the 125 mg/70 kg (*p* < .001) and 250 mg/70 kg (*p* = .004) dose conditions. Though not statistically significant, mean intensity values were notably higher for the 125 mg/70 kg dose^1^ (321.91; *SE* = 112.60) than for the 250 mg/70 kg dose (182.27; *SE* = 91.85; *p* = .17); after adjusting for unit price, the difference in intensity across the active doses was markedly reduced, where participants purchased only slightly more of the 250 mg/70 kg dose than the 125 mg/70 kg dose; the difference remained not statistically significant, *t*(10) = −0.04, *p* = .97*, g* = −.01. There was not a significant difference in the *α* parameters^2^ between the 125 mg/70 kg (0.001; *SE* = 0.0007) and 250 mg/70 kg doses (0.002; *SE* = 0.0008), *t*(10) = −0.14, *p* = .89, *g* = −.04; after adjusting for unit price (shared *k* = 3.00), *α* values were lower (i.e., more persistent consumption as price increased) for the 250 mg/70 kg dose, though this difference was not statistically significant, *t*(10) = 0.22, *p* = .83, *g* = .06.

Equation 1 provides a moderate-to-good description of the purchasing data for the methamphetamine study across the 20 mg (*R*^2^ = .70, *RMSE* = .17) and 40 mg (*R*^2^ = .93, *RMSE* = .14) doses. Data from one participant were identified as nonsystematic based on the first criterion and an additional case was determined to be an extreme outlier (intensity_20 mg_ = 10,000; *z* = 3.87). Both cases were excluded from further analyses. One participant reported invariant consumption across all prices for the 20 mg dose and was thereby excluded only from analyses of the *α* parameter. There was a significant main effect of dose on intensity (*F*_2,32_ = 8.06, *p* = .002, ${\eta}_{\textrm{p}}^2$ = .34), where there were significantly lower values for the placebo than for the 20 mg (*p* = .001) and 40 mg (*p* = .002) doses. Though not statistically significant, there was less purchasing of the 20 mg dose (59.64; *SE* = 23.10) than the 40 mg dose (70.82; *SE* = 37.12; *p* = .98); interestingly, a unit-price analysis indicated greater purchasing of the 40 mg dose than would be expected based on unit price of the dose alone; this difference, however, was not statistically significant, *t*(16) = -1.69, *p* = .11, *g* = −.40. There were no significant differences in the *α* parameter values between the 20 mg dose and the 40 mg dose (*t*_15_ = 0.22, *p* = .83, *g* = .05); however, significantly lower *α* values emerged for the 40 mg dose in the unit-price analysis (shared *k* = 1.86; *t*_15_ = 2.41, *p* = .03, *g* = .59), indicating more persistent consumption for this dose.

Lastly, purchasing data for the alcohol study were also well described by Eq. 1 (*R*^2^ = .95, *RMSE* = .15). Data from 4 participants were identified as nonsystematic based on the first criterion and excluded from further analyses. Comparisons within the alcohol group were restricted to a pairwise analysis of intensity, where this group reported significantly lower values for the placebo condition than for the 1.0 g/kg condition, *t*(20) = −4.38, *p* < .001, *g* = −.87.

### Subjective drug effects

There was a significant main effect of dose on peak drug “liking” in the cocaine study (*F*_2, 20_ = 85.53, *p* < .001, ${\eta}_{\textrm{p}}^2$ = .90), where ratings were lower for the placebo than for the 125 mg/70 kg (*p* < .001) and 250 mg/70 kg (*p* < .001) doses. The difference between the two active doses was also significant, with higher ratings for the 250 mg/70 kg dose than for the 125 mg/70 kg dose (*p* = .002). Peak “good effects” of the drug also differed as a function of dose (*F*_2,20_ = 75.08, *p* < .001, ${\eta}_{\textrm{p}}^2$ = .88), with lower ratings for the placebo than the 125 mg/70 kg (*p* < .001) and 250 mg/70 kg (*p* < .001) doses. Again, ratings were significantly higher for the 250 mg/70 kg dose than for the 125 mg/70 kg dose (*p* = .001).

In the methamphetamine study, peak drug “liking” also significantly differed as a function of dose (*F*_2,32_ = 31.52, *p* < .001, ${\eta}_{\textrm{p}}^2$ = .66), with significantly lower ratings for the placebo than for the 20 mg (*p* < .001) and 40 mg (*p* < .001) doses. Differences between the 20 mg and 40 mg doses were not significant (*p* = .45), though ratings for the 40 mg dose were marginally higher than for the 20 mg dose. There was also a significant effect of dose on peak “good effects” of the drug (*F*_2,32_ = 22.18, *p* < .001, ${\eta}_{\textrm{p}}^2$ = .58), where lower ratings occurred for the placebo than for the 20 mg (*p* < .001) and 40 mg doses (*p* < .001). Ratings were marginally higher for the 40 mg dose than for the 20 mg dose, yet were not statistically significant (*p* = .72).

Finally, in the alcohol study, there were significantly lower ratings of peak “liking” for the placebo than for the 1.0 g/kg active dose (*t*_20_ = −5.32, *p* < .001, *g* = −1.14), along with significantly lower peak ratings of “good effects” for the placebo than for the 1.0 g/kg dose, *t*(20) = −4.89, *p* < .001, *g* = −1.05.

### Bivariate associations between behavioral economic demand measures, substance use patterns, and subjective drug effects

Correlations describing the degree to which purchase task indices were associated with reported substance use patterns and peak subjective drug effects can be found in Table 2. For the cocaine study, there were robust positive associations between *O*_max_ for the 125 mg/70 kg dose and money spent on cocaine per week (*r* = .63, *p* =.04), as well as the number of days cocaine was used in the last month (*r* = .61, *p* = .045). Similar associations with *O*_max_ emerged for the 250 mg/70 kg dose (money spent per week: *r* = .61, *p* = .048; number of days used in the last month: *r* = .64, *p* = .03). There were also significant associations between peak subjective effects and demand for the 250 mg/70 kg dose, though higher peak ratings were generally associated with less consumption. For example, participants who reported higher peak “liking” of the 125 mg/70kg dose tended to have lower *P*_max_ values (*ρ* = −.63, *p* = .041) and those reporting higher peak “good effects” of the 250 mg/70 kg dose tended to have lower intensity values (*ρ* = −.63, *p* = .042). This may have been a function of individual differences in drug tolerance, as suggested by the inverse trend between money per week spent on cocaine and ratings of “good effects” for the 250 mg/70kg dose (*ρ* = −.59, *p* = .06).

**Table 2:** Bivariate associations between behavioral economic indices, substance use patterns, and peak subjective effects

Study	Index	Dose	Pearson *r*			Spearman *ρ*	
			Money spent/week	Times used/week	Days used/month	Peak “liking”	Peak “good effects”
Cocaine	Intensity	125	.13	.35	.36	.46	.31
		250	.16	−.09	.05	−.34	−.63*
	*α*	125	−.20	−.11	−.05	−.31	−.31
		250	−.22	−.43	−.40	.18	.08
	*O*_max_	125	.63*	.58	.61*	.01	−.09
		250	.61*	.52	.64*	−.49	−.41
	*P*_max_	125	.30	−.04	.09	−.63*	−.58
		250	−.14	−.24	.03	.08	.45
	*BP*_1_	125	−.07	−.31	−.17	.06	.18
		250	−.16	−.22	−.06	.20	.33
Methamphetamine	Intensity	20	.56*	.00	.06	.05	−.09
		40	.49*	−.06	.04	.09	−.01
	*α*	20	.08	−.24	.04	−.10	−.04
		40	−.05	−.11	.14	−.14	−.07
	*O*_max_	20	.52*	.56*	.47	−.16	−.39
		40	.60*	.42	.26	.06	−.09
	*P*_max_	20	.33	.53*	.42	−.15	−.39
		40	.59*	.61**	.42	−.02	−.21
	*BP*_1_	20	.25	.49*	.34	−.15	−.26
		40	.42	.45	.20	.19	.02
Alcohol	Intensity	1.0	.43*	.48*	.39	.29	.30
	*α*	1.0	−.31	−.29	−.18	−.34	−.34
	*O*_max_	1.0	.34	.26	.17	.32	.33
	*P*_max_	1.0	.23	.07	.02	.12	.35
	*BP*_1_	1.0	.25	.15	.09	.31	.52*

Drug doses are reported as mg/70 kg for cocaine, mg for methamphetamine, and g/kg for alcohol

*p ≤ .05; ** *p* ≤ .01

In the methamphetamine study, the amount of money spent on stimulants each week was significantly associated with intensity across the 20 mg (*r* = .56, *p* = .02) and 40 mg (*r* = .49, *p* = .048) doses. Significant associations also emerged between the amount of money spent on stimulants each week and *O*_max_ across the 20 mg (*r* = .52, *p* = .03) and 40 mg (*r* = .60, *p* = .01) doses as well as *P*_max_ for the 40 mg dose (*r* = .59, *p* = .01). Weekly stimulant use was associated with *O*_max_ for the 20 mg dose (*r* = .56, *p* = .02); *P*_max_ for the 20 mg (*r* = .53, *p* = .03) and 40 mg (*r* = .61, *p* = .01) doses; and *BP*_1_ for the 20 mg dose (*r* = .49, *p* = .05). As was the case in the cocaine study, peak subjective effects were inversely associated with substance use patterns. Here, peak “liking” was inversely associated the number of days stimulants were used in the last month across the 20 mg (*ρ* = −.62, *p* = .01) and 40 mg (*ρ* = −.65, *p* = .01) doses; peak “good effects” were also inversely associated with the number of days stimulants were used in the last month across the 20 mg (*ρ* = −.66, *p* = .01) and 40 mg (*ρ* = −.65, *p* = .01) doses as well as money spent on stimulants per week across the 20 mg (*ρ* = −.55, *p* = .03) and 40 mg (*ρ* = −.55, *p* = .03) doses.

Finally, in the alcohol study, significant associations emerged between intensity and money spent on alcohol each week (*r* = .43, *p* = .05) and weekly alcohol use (*r* = .48, *p* = .03).

## Discussion

Across all substance administration studies, the Blinded-Dose Purchase Task yielded orderly demand data that showed significantly greater demand in the active dose conditions compared to the placebo conditions using a double-blind, placebo-controlled within-subject design. Demand was significantly correlated with substance use and monetary patterns of drug spending, as well as some subjective effects of the drugs. These relations differed by drug and dose administered. These data highlight the potential of the Blinded-Dose Purchase Task for assessing drug demand while controlling for drug expectancy effects.

The results underscore the viability of this task as an addition to laboratory drug administration studies. The task takes approximately 3–5 min to administer, offering a simple and straightforward assessment that provides additional information on drug reinforcement beyond what is typically provided by a typical abuse liability study that only assesses subjective effects. The present findings significantly extend results from purely hypothetical purchase tasks, and represent an important preliminary step in extending the literature. Specifically, the present studies and analyses build on a noteworthy study in which stimulant-naïve individuals completed a blinded purchase task following a single dose of D-amphetamine or placebo (MacKillop et al., 2019). Compared to placebo, the single dose of D-amphetamine significantly increased intensity, breakpoint, and *O*_max_, and decreased elasticity, while *O*_max_ and breakpoint mediated the relationship between subjective drug effects and willingness to take the drug again (MacKillop et al., 2019). In each of the present experiments, simulated consumption of the experienced substances was orderly and varied as an inverse function of escalating price, with good to excellent fits provided by Eq. 1. All active dose conditions compared to placebo also showed significantly higher purchasing when the drug was inexpensive (intensity). Despite these methodological extensions, neither study (MacKillop et al., 2019; the present study) compared the results of a purely hypothetical purchase task to a blinded experiential purchase task. A critical next step and area for future research will be to determine how the purely hypothetical drug purchase tasks track blinded experiential tasks, with potential similar or divergent results.

Contrary to previous work, in the present study, we used the novel Blinded-Dose Purchase Task to assess reinforcing value of the dose effects of cocaine and methamphetamine and a single dose of alcohol, *while controlling for drug expectancies in populations that had familiarity with the drug or drug class and varying levels of use* (e.g., recreational alcohol use, cocaine dependence). Hypothetical drug purchase tasks have rarely accounted for drug expectancies, which are thought to impact a wide range of outcomes across numerous drug classes (Berna et al., 2017; Chermack & Taylor, 1995; Jaffe & Kilbey, 1994; Leventhal & Schmitz, 2006; Maganaris et al., 2000; Schafer & Brown, 1991; Testa, 2002; Volkow et al., 2003). For example, Aston et al. (2021) used qualitative methods to inform the design of a hypothetical cannabis purchase task. Participant responses included comments that highlighted the different quality of cannabis as problematic in the task where they were asked to imagine cannabis of “average” quality and strength (e.g., “average quality sounds like worse than what I would usually expect,” pg. 27, or … “your average might not be the same as my average,” pg. 27; Aston et al., 2021). Again, future research could directly compare demand across the Blinded-Dose Purchase Task and a purely hypothetical purchase task to understand potential differences and the role expectancy effects play in standard hypothetical purchase tasks.

The demand metrics evaluated appear to track real-world drug spending and drug use behavior, further highlighting the viability of the present task. In the cocaine administration study in which individuals who met *DSM-IV* criteria for cocaine abuse or dependence participated, cocaine spending (money spent on cocaine in the past week) and self-reported cocaine use (number of days used cocaine in the last month) were strongly and significantly positively correlated with *O*_max_ for both cocaine doses. These results align with a previous study evaluating a purely hypothetical Cocaine Purchasing Task among individuals who also met *DSM-IV* criteria for cocaine dependence (Bruner & Johnson, 2014), in which significant and strong positive correlations were observed between *O*_max_ and self-reported measures of cocaine use (money spent on cocaine per week and days of cocaine use in the past month). As predicted by the behavioral economic framework in which lower compared to higher active doses result in greater consumption, simulated cocaine purchasing was moderately (and non-significantly) higher when inexpensive (intensity) for the 125 mg/70 kg dose compared to the 250 mg/70 kg dose. After adjusting for unit price, however, the difference in intensity as expected was notably reduced. No differences were observed in the *α* parameter (rate of change of elasticity) across active lower and higher cocaine doses, although unit-price analyses revealed purchasing of 250 mg/70 kg dose compared to the lower 125 mg/70 kg dose to be marginally more persistent across increases in price (see further below for a discussion of unit-price study findings).

Higher peak ratings in the 250 mg/70 kg cocaine dose condition were generally associated with less persistent consumption. Specifically, participants who reported higher peak subjective ratings also tended to report lower intensity and marginally lower *O*_max_. A similar pattern emerged between peak ratings in the 125 mg/70 kg dose condition and *P*_max_. It is possible that these relations could represent individual differences in tolerance, as lower “liking” or “good” effects of cocaine experienced may require increased cocaine purchasing to maintain desired effects. The Blinded-Dose Purchase Task allows for evaluation of relations between subjective drug effects experienced and demand, which would not be possible in a standard hypothetical purchase task.

In the methamphetamine administration study, in which individuals who recreationally used stimulants participated, money spent on stimulants each week was significantly and positively associated with demand metrics further validating this task. Specifically, money spent on stimulants each week was significantly and positively associated with intensity and *O*_max_ for the 20 and 40 mg doses as well as with *P*_max_ for the 40 mg dose. Significant positive associations were also observed between weekly stimulant use and *P*_max_ for the 20 and 40 mg doses along with *O*_max_ and *BP*_1_ for the 20 mg dose. Although hypothetical methamphetamine purchase tasks have rarely been investigated (Chalmers et al., 2010; Yoon et al., 2021), results of the present study are generally consistent with previous research, although somewhat divergent. Yoon et al. (2021) assessed simulated methamphetamine purchasing among individuals with methamphetamine use disorder and found significant positive correlations between intensity for methamphetamine and self-reported methamphetamine used in the past month, but no other demand metrics utilizing standard correlations as in the present study. These differences may be due to varied methods used in the demand task (drug had to be consumed that day in Yoon et al., versus 1 month in the present study) or population (individuals with methamphetamine use disorder versus recreational stimulant users).

Simulated methamphetamine purchasing did not appear to show indications of dose effects for the 20 mg or 40 mg doses for either intensity or rate of change of elasticity (*α*), and these results generally align with the peak subjective effect data (no significant difference between 20 and 40 mg peak subjective effect data, although see Berry et al., 2022, for additional subjective effect data). It is possible that the lack of difference between simulated purchasing of methamphetamine and subjective effects is related to variability in responses, as well as the somewhat high doses administered. A greater differential between dose effects of simulated purchasing (and subjective effects) may be more robust at a decreased “low” dose (e.g., 10 mg low dose compared to 20 mg high dose) as opposed to the somewhat higher doses used in the present study (20 mg as the low dose versus 40 mg as the high dose). The high dose of methamphetamine (40 mg) was originally selected due to misuse occurring at higher doses in this range among stimulant users, and has also been safely administered to individuals who use stimulants in previous studies (e.g., Marone et al., 2010), and the lower dose (20 mg) was selected as half the high dose to examine potential dose effects.

Unit-price analyses indicated somewhat higher intensity and significantly lower *α* (greater persistence in purchasing with increases in price) for the 40 mg dose compared to the 20 mg dose, indicating greater consumption of the 40 mg dose than would be expected based solely on the unit price. The behavioral economic framework suggests that consumption should be the same if the unit price is the same (Bickel et al., 1990). This difference in rate of change of elasticity (*α*) may be related to the hypothetical nature of the task. It is possible that real money spent on real doses received would yield convergence in rate of change of elasticity across doses. These results highlight the importance of unit-price evaluations of multiple doses for comparison across demand metrics (e.g., rate of change of elasticity, Hursh and Silberberg, 2008), revealing differences that may otherwise be overlooked. Unit-price analyses also allow for parsimonious comparisons across studies (Bickel, et al. 1990; Bickel et al., 1991; Hursh & Silberberg, 2008) and assessments of behavioral or pharmacological manipulations across doses. This Blinded-Dose Purchase Task lends itself to comparisons of multiple doses more generally and unit-price analyses more specifically than typical purely hypothetical purchase tasks, which often employ a single average dose.

Similar to the cocaine study, peak subjective effects for methamphetamine at the 20 and 40 mg doses were inversely associated with money spent on stimulants per week and the number of days stimulants were used in the last month, potentially representing tolerance with increased purchasing with decreased “liking” or “good” effects. These results highlight the importance of multidimensional evaluations of drug reinforcement when administering substances. More research is needed in this regard to understand drug administration in the context of dose effects of hypothetical purchasing and relations to subjective effects, as well as other informative demand metrics that may illuminate clinically relevant drug purchasing behaviors (e.g., understanding drug purchasing in the context of commonly co-used drugs, cross-price elasticity; Bergeria et al., 2020).

In the alcohol administration study, in which individuals who reported at least occasionally drinking 4–5 drinks per episode participated, significant positive associations emerged between intensity and money spent on alcohol per week and weekly alcohol use as well as between *BP*_1_ and peak “good” effects. A recent systematic review and meta-analysis of the alcohol purchase task (Martínez-Loredo et al., 2021) suggested that intensity may be one of the most relevant indices to account for alcohol use, hazardous drinking, and heavy drinking. Although we did not measure hazardous drinking in the present study, the associations between intensity and weekly alcohol use align with the findings of the meta-analysis. Furthermore, peak subjective effects in the alcohol study were positively associated with *BP*_1_, showing that “good” effects may result in more persistent purchasing for alcohol as measured by *BP*_1_. A limitation of the alcohol study is that dose effects were not assessed, and this will be an important area of future research.

Data from the present studies generally align with purely hypothetical tasks in which the doses are not administered, highlighting the versatility and viability of purchase tasks. However, more research is needed to understand potential differences of purchase task metrics across different drugs, doses, and populations. Far less research exists regarding methamphetamine or cocaine purchase tasks relative to alcohol purchase tasks, making conclusions as to the most critical and predictive demand metrics and how these might differ across drugs/drug classes difficult (see Martínez-Loredo et al., 2021, for discussion of utility of different demand metrics for alcohol related outcomes). By examining multiple metrics (e.g., intensity, elasticity), demand analyses offer an advantageous multidimensional evaluation of drug reinforcement compared to traditional approaches that often treat reinforcement as a homogenous concept (Hursh & Silberberg, 2008; Johnson & Bickel, 2006). This task employed in the present studies lays the groundwork for methods that can be used to test the abuse potential of novel or familiar compounds. For example, future research could use this task to control for drug expectancies and evaluate multiple demand metrics to evaluate the risk of abuse potential for a particular drug, unfamiliar drug combinations, novel substances among experienced users, or interventions designed to reduce drug abuse potential (MacKillop et al., 2019), as well as assess relations between traditional subjective drug effects experienced and demand.

As with any study, these findings should be considered in the context of study limitations. These studies and analyses should be considered preliminary, but represent an important first step in evaluating forced sampling methods in cocaine, methamphetamine, and alcohol purchase tasks. An important future direction will be to directly compare purchasing results of forced sampling methodology as used in the present study, with results obtained from purely hypothetical purchase tasks with cocaine, methamphetamine, and alcohol purchasing. Currently, purely hypothetical purchase tasks are much more common in the literature (although see Bergeria et al., 2019; Cassidy et al., 2019; Higgins et al., 2018, for forced sampling Cigarette Purchase Task examples), with limited comparisons to purchasing under forced sampling methods. Similar findings resulting from direct comparisons would lend confidence to both approaches. Our results also map on, in part, to subjective drug effects and real-world drug spending, lending additional confidence to our findings and establishing initial relations to be examined in future studies.

Furthermore, the sample size was limited and a portion of the findings are correlational in nature. Increased statistical power upon replication would allow for greater flexibility in analyses and increased credence to the present findings. Multi-level modeling which can incorporate multiple variables of interest into a single analysis (e.g., Kaplan et al., 2021) might be used in future studies, in addition to an increased sample size. The subjective drug effects questionnaires used a 5-point scale, rather than a 100-mm VAS format. The 5-point scale, however, has been used in previous research and appears to produce comparable results to the 100-mm VAS scale (e.g., Johnson et al., 2017). Nevertheless, the scale could potentially limit the distribution of responses. Furthermore, while the purchase task was administered at the end of the session so participants could experience the full time course of the drugs, results may differ if the purchase task was delivered during peak drug effects (e.g., drug administration can increase drug purchasing, e.g., Amlung et al., 2015b). However, we attempted to avoid immediate drug priming-type effects that could result in increased purchasing by administering these tasks toward the end of the session, when peak effects had diminished. Future research might investigate the effects of the timing of administration of the Blinded-Dose Purchase Task in relation to drug administration. Although such future research is important for continued validation of the task, the present and previous researches indicate that the Blinded-Dose Purchase Task may provide a powerful tool for rigorously investigating the behavioral economics of drug effects and abuse liability.

## Supplementary information

ESM 1 (DOCX 817 kb)

## Footnotes

1 Raw values used for descriptive reporting and transformed values for statistical tests.

2 Analyses involving α parameters were restricted to the active doses due to the high proportion of participants reporting “0” across all prices for the placebo, thereby precluding model fitting at the individual level.
