# Supplementary material for: The Blinded-Dose Purchase Task: assessing hypothetical demand based on cocaine, methamphetamine, and alcohol administration
Source: Psychopharmacology (Berl). 2023 Mar 4;240(4):921–33. doi: 10.1007/s00213-023-06334-6 (PMC10006272; doi:10.1007/s00213-023-06334-6)
Supplement: Supplementary file 1 — (DOCX 817 kb) [file 213_2023_6334_MOESM1_ESM.docx]

**Supporting Materials**

**
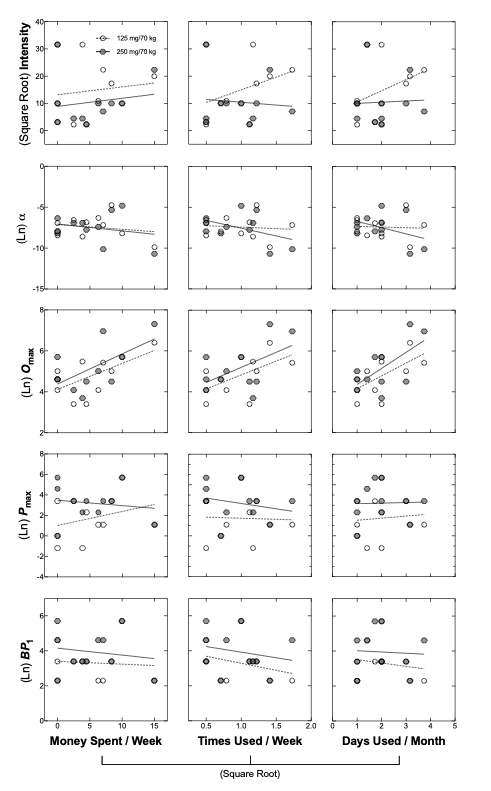
**

Figure S1. Pearson correlations between Blinded-Dose Purchase Task demand indices and reported real-world cocaine consumption. See manuscript (Table 2) for correlation coefficients and statistical significance.

**
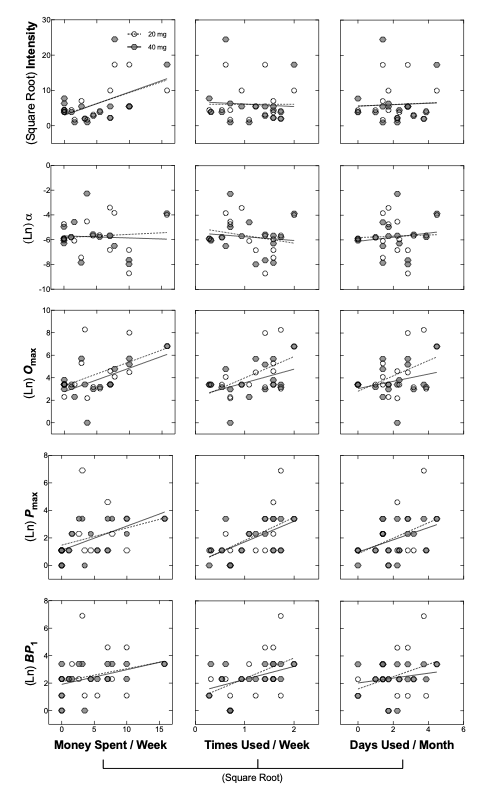
**

Figure S2. Pearson correlations between Blinded-Dose Purchase Task demand indices and reported real-world stimulant consumption. See manuscript (Table 2) for correlation coefficients and statistical significance.

**
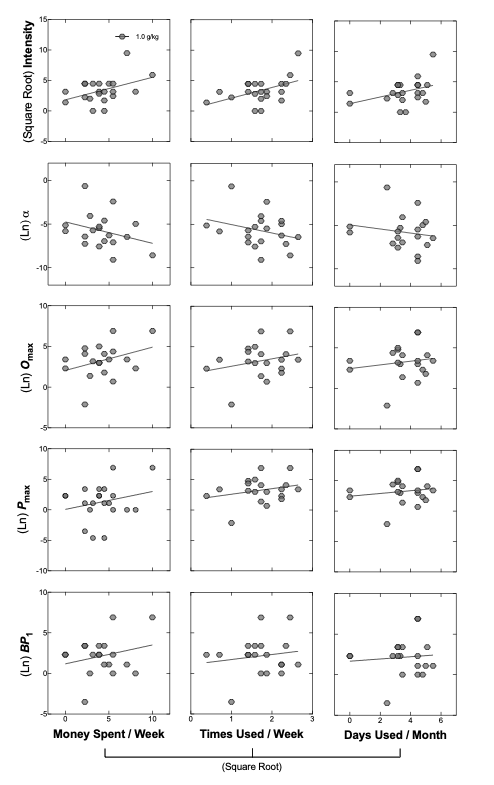
**

Figure S3. Pearson correlations between Blinded-Dose Purchase Task demand indices and reported real-world alcohol consumption. See manuscript (Table 2) for correlation coefficients and statistical significance.
